# Selectively targeting haemagglutinin antigen to chicken CD83 receptor induces faster and stronger immunity against avian influenza

**DOI:** 10.1038/s41541-021-00350-3

**Published:** 2021-07-15

**Authors:** Angita Shrestha, Jean-Remy Sadeyen, Deimante Lukosaityte, Pengxiang Chang, Adrian Smith, Marielle Van Hulten, Munir Iqbal

**Affiliations:** 1 The Pirbright Institute, Pirbright, Woking, Surrey, United Kingdom; 2 Department of Zoology, Peter Medawar Building, University of Oxford, Oxford, United Kingdom; 3 Global Poultry R&D Biologicals Boxmeer, Intervet International BV, MSD Animal Health, Boxmeer, The Netherlands

**Keywords:** Vaccines, Vaccines, Virology

## Abstract

The immunogenicity and protective efficacy of vaccines can be enhanced by the selective delivery of antigens to the antigen-presenting cells (APCs). In this study, H9N2 avian influenza virus haemagglutinin (HA) antigen, was targeted by fusing it to single-chain fragment variable (scFv) antibodies specific to CD83 receptor expressed on chicken APCs. We observed an increased level of IFNγ, IL6, IL1β, IL4, and CxCLi2 mRNA upon stimulation of chicken splenocytes ex vivo by CD83 scFv targeted H9HA. In addition, CD83 scFv targeted H9HA induced higher serum haemagglutinin inhibition activity and virus neutralising antibodies compared to untargeted H9HA, with induction of antibodies as early as day 6 post primary vaccination. Furthermore, chickens vaccinated with CD83 scFv targeted H9HA showed reduced H9N2 challenge virus shedding compared to untargeted H9HA. These results suggest that targeting antigens to CD83 receptors could improve the efficacy of poultry vaccines.

## Introduction

Vaccines are major tools to reduce the devastating impact of infectious disease in farmed animals and humans. However, many vaccines are administered in multiple doses, inducing sub-optimal immunity that might protect from clinical disease and death, but does not prevent shedding of infectious pathogens from vaccinated animals. Thus, the endemic cycle of disease continues. The challenge is to improve the effectiveness of vaccines to reduce transmission and increase protection from disease by inducing strong and long-lasting humoral and cellular immunity. In recent years, various strategies have been developed to enhance the immunogenicity of vaccines. One such strategy is the recombinant targeted antigen delivery vaccine (TADV) whereby protective antigens are selectively delivered to professional antigen-presenting cells (APCs) such as dendritic cells (DC), macrophages, and B cells^1^. These cells capture, process, and present antigens to T lymphocytes for initiation and maintenance of immune responses. At present, either pattern recognition receptors (PRRs) agonist-or antibody-based conjugates, are used for targeting antigens to APCs. In PRR agonist-based APC targeting, antigens are conjugated to molecules such as lipopolysaccharide (LPS), DNA containing unmethylated CpG motifs, or flagellin whereas in antibody-based targeting, antigens are conjugated to antibodies that recognise specific receptors on APCs^2^. Targeting antigens to APCs using antibody-based methods can be carried out by either chemically conjugating the antigen to a monoclonal antibody (mAb) specific for selected APC receptors or by genetic engineering in which the antigen is fused to antibody fragments such as single-chain fragment variable (scFv) antibodies specific for the APC receptors^3–5^. The scFv antibodies have been popular for their use in antigen targeting due to their small size. They are the smallest unit of immunoglobulin molecule that holds a complete antigen-binding domain of an antibody and are 25−30 kDa in size^6,7^. They can be synthetically generated by connecting the variable domains (vH and vL) with a flexible short (16−20 amino acids) peptide linker. The scFv antibodies retain the binding specificity of the parent antibody and provide several advantages compared to the complete mAb IgG molecule containing the fragment crystalisable (Fc) region, in various therapeutic and diagnostic applications^7^. When antigens are targeted to specific receptors on APCs, their non-specific uptake can be reduced. This can increase the quantity of the antigens reaching the APCs.

Previously, various studies explored antibody-based antigen targeting mammalian APCs via DC receptor for endocytosis-205 (Dec205)^8–10^. The first antigen-targeting study in chickens was also directed towards Dec205 receptor where the complete haemagglutinin (HA) protein of the H5N2 virus was chemically conjugated to anti-chicken Dec205 mAb. A single dose of this vaccine was shown to be sufficient to elicit a strong antibody response in chickens as early as fourteen days after priming^10^. Furthermore, targeting a synthetic peptide antigen to chicken CD40 receptor showed accelerated and enhanced antibody responses against the peptide antigen compared to untargeted peptide^11^. PRR-agonist-based targeting has also been investigated in chickens before. Song et al. generated a H7HA influenza subunit vaccine recombinantly fused to *Salmonella typhimurium* flagellin (H7HA-fliC). Immunisation of chickens with H7HA-fliC showed robust antibody responses leading to a significant reduction in viral loads compared to the chickens receiving only H7HA^12^. Various other receptors like CD11c, CD80, Clec9A, and MHC II have been used in mammals for antibody-based antigen targeting^13–19^. However, these APC receptors have not been examined in chickens for antigen targeting. One potential receptor molecule which has not yet been explored for antigen targeting in either the mammalian or the avian system is CD83. In mammals, CD83 is a surface glycoprotein that belongs to the immunoglobulin superfamily. CD83 is predominantly expressed on DCs and is an early activation marker for DCs^20^. However, CD83 is also expressed in activated macrophages, natural killer cells, and activated T and B lymphocytes^21^. CD83 has been thought to be involved in immune response; however, its function on DCs and T cells remains unclear. Based on the expression profile of CD83 and its structural similarity with B7 family members (CD80/CD86), CD83 is thought to play important roles during interactions between cells of the immune system^22^. Recently, it was reported that CD83 plays a major role in B cell function for antibody production, in response to influenza A virus infection^23^. Moreover, chicken CD83 has been characterised, and was shown to have 39% and 40% amino acid sequence similarity to human and mouse CD83, respectively^24^.

There are limited data available for targeting chicken APCs for modulating immunogenicity of poultry vaccines^10^. Here, we provide evidence that targeting chicken CD83 enhances the immunogenic potential of antigens by inducing faster and stronger immune responses. In this study, we selected avian influenza virus (AIV) H9N2 haemagglutinin (HA) as a model antigen reconstituted as a recombinant subunit vaccine. The HA protein lacking transmembrane domain (TM) was fused to scFv antibodies specific for chicken CD83 receptors and produced as a soluble trimeric protein in *Drosophila melanogaster* S2 cells. To the best of our knowledge, this is the first report of CD83 receptor, being used for antigen targeting studies. This strategy could be used to develop new and effective vaccines against several infectious animal and human diseases.

## Results

### Expression and purification of the recombinant proteins

**Fig. 1:**
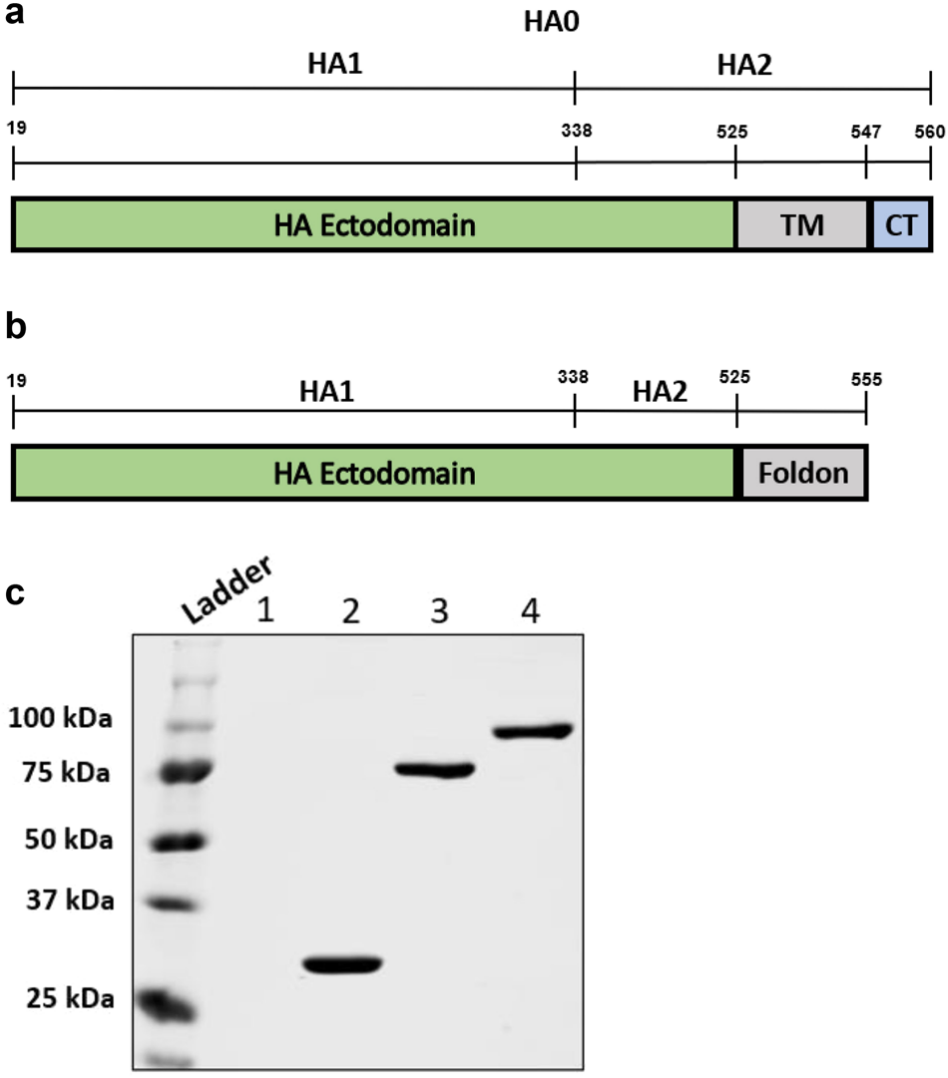
Construction and purification of recombinant HA proteins. **a** Schematic representation of the full length HA protein of H9N2 (MsCon) virus. Precursor HA0 :1-560 amino acids (aa), HA1:19-338 aa, HA2: 339-560 aa, TM = transmembrane domain (525-547 aa) CT = cytosolic tail domain (548−560 aa). **b** Schematic representation of the soluble HA protein of H9N2 (MsCon) virus. The soluble H9HA was generated by removing the TM and CT domains (525-560 aa) and fusing the C-terminus of HA to 30 aa long trimerisation foldon sequence of the trimeric protein fibritin from T4 bacteriophage **c** His tag purification of the recombinant proteins. The expected sizes of the purified CD83 scFv antibody, rH9HA, and rH9HA-CD83 scFv are 30, 70, and 100 kDa respectively. Lane 1: control supernatant from the untransfected cells Lane 2: CD83 scFv Lane 3: rH9HA Lane 4: rH9HA-CD83 scFv. For the purification of the recombinant proteins, the harvested S2 cell culture supernatants containing recombinant protein bound to the metal ions (copper sulfate was used as an inducer of metallothionein promoter) were loaded onto uncharged “UNOsphere” resin derivatized with iminodiacetic acid functioning as a chelating ligand (Profinity^™^IMAC, Bio-Rad). Proteins were eluted with elution buffer containing 50 mM NaH_2_PO_4_, 300 mM NaCl and 50 mM imidazole. The purified proteins were analysed by 10% SDS-PAGE followed by Coomassie staining. All blots were derived from the same experiment and were processed in parallel.

To create a soluble H9HA protein, the TM domain of H9HA was replaced with the 30 amino acid long foldon of the trimeric protein fibritin from bacteriophage T4 (hereinafter referred to as rH9HA, Fig. 1a, b). Furthermore, rH9HA was fused to scFv antibody targeting the CD83 receptor protein on chicken APCs (hereinafter referred to as rH9HA-CD83 scFv). The CD83 scFv, rH9HA, and rH9HA-CD83 scFv proteins were expressed in *Drosophila melanogaster* S2 cells. Subsequent purification of the recombinant proteins by His-tag affinity chromatography produced proteins with the expected molecular weights of about 30 kDa for CD83 scFv, 70 kDa for rH9HA, and 100 kDa for rH9HA-CD83 scFv (Fig. 1c). For rH9HA and rH9HA-CD83 scFv proteins, a single polypeptide of about 70 and 100 kDa respectively was observed on SDS-PAGE under reducing conditions. This indicates that the recombinant rH9HA protein is expressed as a HA precursor (HA0). Based on recovered purified proteins, it was estimated that expression levels of recombinant proteins ranged from 10 to 20 mg/litre of culture supernatant.

### H9HA ectodomain fused to T4 bacteriophage foldon can trimerise and retain haemagglutination activity

**Fig. 2:**
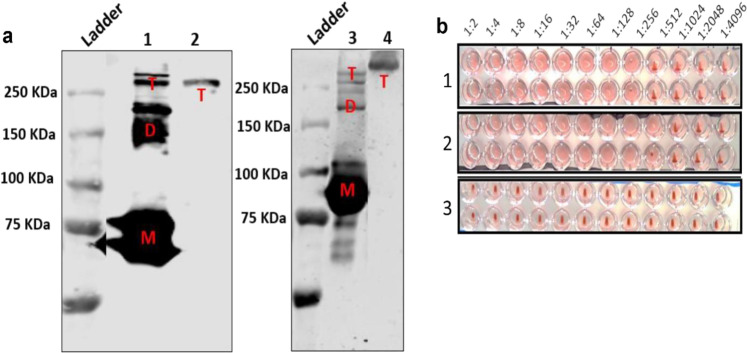
Analysis of the oligomeric form and activity of recombinant H9HA ectodomain containing foldon. **a** BS3 cross-linking experiment for determining the native structure of H9HA ectodomain with foldon. Lane 1: rH9HA without BS3 Lane 2: rH9HA with 10 mM BS3 Lane 3: rH9HA-CD83 scFv without BS3 Lane 4: rH9HA-CD83 scFv with 10 mM BS3. About 15 µg of the recombinant protein was mixed with BS3 to a 10 mM final concentration and incubated for 1 h at room temperature. The cross-linking reaction was stopped by the addition of 1 M Tris-HCl pH 8.0 to a final concentration of 50 mM and incubated for 15 min at room temperature. After cross-linking, proteins were separated on 8% SDS-PAGE under reducing conditions, blotted, and analysed by western blot using anti-H9HA monoclonal antibody. M: monomer (70 kDa* 100 kDa^) D: dimer (140 kDa* 200 kDa^) T: Trimer (210 kDa* 300 kDa^) *rH9HA ^rH9HA-CD83 scFv. All blots were derived from the same experiment and were processed in parallel. **b** Haemagglutination assay to test the activity of recombinant H9HA with foldon to agglutinate chicken red blood cells. 1. rH9HA 2. rH9HA–CD83 scFv 3. Negative control (PBS). For the haemagglutination assay, a two-fold serial dilution of 35 μg of the recombinant HA proteins was carried out in 96-well plates. About 50 μl of 1% chicken RBCs was added. The plates were incubated at 4 °C for 1 h and the highest dilution of the protein causing the agglutination of the RBCs was noted.

The oligomerisation state of soluble H9HA protein with trimerisation foldon was determined by cross-linking using Bis[sulfosuccinimidyl] suberate (BS3). Multimeric proteins exposed to this cross-linker will have each subunit cross-linked together with the formation of amide bonds^25^. This provides direct evidence for their close proximity^26^. This also helps to stabilise the structure of oligomers, allowing them to be resolved on SDS denaturing gels for western blot analysis^25^. This method was used to confirm the native structure of the protein. Recombinant rH9HA and rH9HA-CD83 scFv proteins were exposed to BS3 cross-linker and the cross-linked products were separated on SDS-PAGE gel under reducing and denaturing conditions, blotted, and immunodetected using anti-H9HA monoclonal antibody. The result is shown in Fig. 2a. Without BS3 crosslinking, three species: monomer, dimer, and trimer were observed (major band at monomer; Lane 1 and Lane 3 corresponding to about 70 and 100 kDa for rH9HA and rH9HA-CD83 scFv, respectively). With crosslinking, the stable trimeric form was observed (Lane 2 and Lane 4 corresponding to about 210 and 300 kDa for rH9HA and rH9HA-CD83 scFv, respectively). This indicates that the native structure of recombinant H9HA protein with foldon is a trimer, and three molecules of scFv antibodies are attached to one trimeric H9HA protein.

Next, the biological activity of soluble rH9HA and rH9HA-CD83 scFv proteins, was tested using haemagglutination assay (Fig. 2b). The soluble rH9HA protein, both on its own and when fused to CD83 scFv antibodies was able to agglutinate chicken red blood cells (RBCs) retaining its haemagglutination activity. An average amount of 0.14 μg of soluble rH9HA and rH9HA-CD83 scFv was required to observe haemagglutination activity.

### The fusion of CD83 scFv antibodies to rH9HA protein does not affect their function

**Fig. 3:**
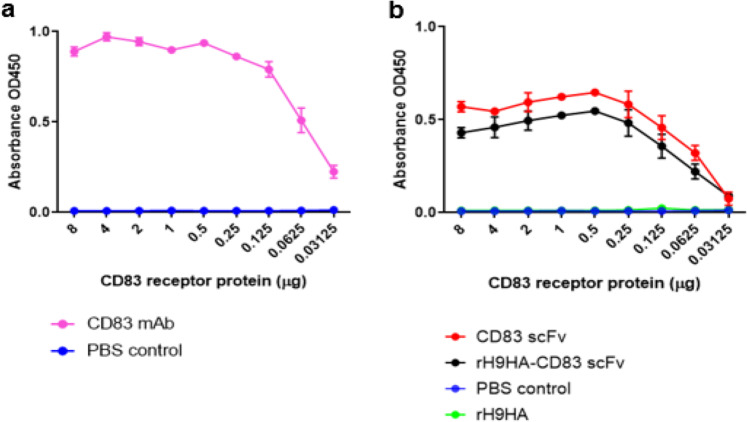
Characterisation of CD83 scFv and rH9HA-CD83 scFv for binding to chicken CD83 ectodomain using ELISA. **a** Indirect ELISA for testing the activity of CD83 mAb **b** Indirect ELISA for testing the activity of CD83 scFv and rH9HA-CD83 scFv. Purified 8 μg of chicken CD83 ectodomain was coated onto each well of the ELISA plate, a two-fold serial dilution was carried out and the plate was incubated overnight for 4 °C. For detection, the plates were incubated with an equimolar concentration of purified CD83 scFv and rH9HA-CD83 scFv or 1 μg/ml of CD83 mAb. This was followed by incubation with goat anti-mouse HRP secondary antibody for (**a**) and HRP-conjugated anti-V5 secondary antibody for (**b**). The colorimetric detection was carried out by adding TMB substrate and absorbance at 450 nm was recorded.

To determine whether the CD83 scFv antibodies can retain their activity after being fused to rH9HA protein, indirect enzyme-linked immunosorbent assay (ELISA) was carried out. Plates were coated with purified chicken CD83 protein (extracellular domain (ED)). CD83 mAb was included in the indirect ELISA assay as a positive control (Fig. 3a). Furthermore, rH9HA was used to assess the unspecific binding of rH9HA to chicken CD83 ED. Moreover, both CD83 scFv and rH9HA-CD83 scFv antibodies were able to detect and bind to chicken CD83 ED (Fig. 3b). Slightly lower binding to chicken CD83 ED by rH9HA-CD83 scFv antibodies was observed, although this was not statistically significant.

### Activation of chicken splenocytes by CD83 scFv, rH9HA, and rH9HA- CD83 scFv proteins ex vivo

**Fig. 4:**
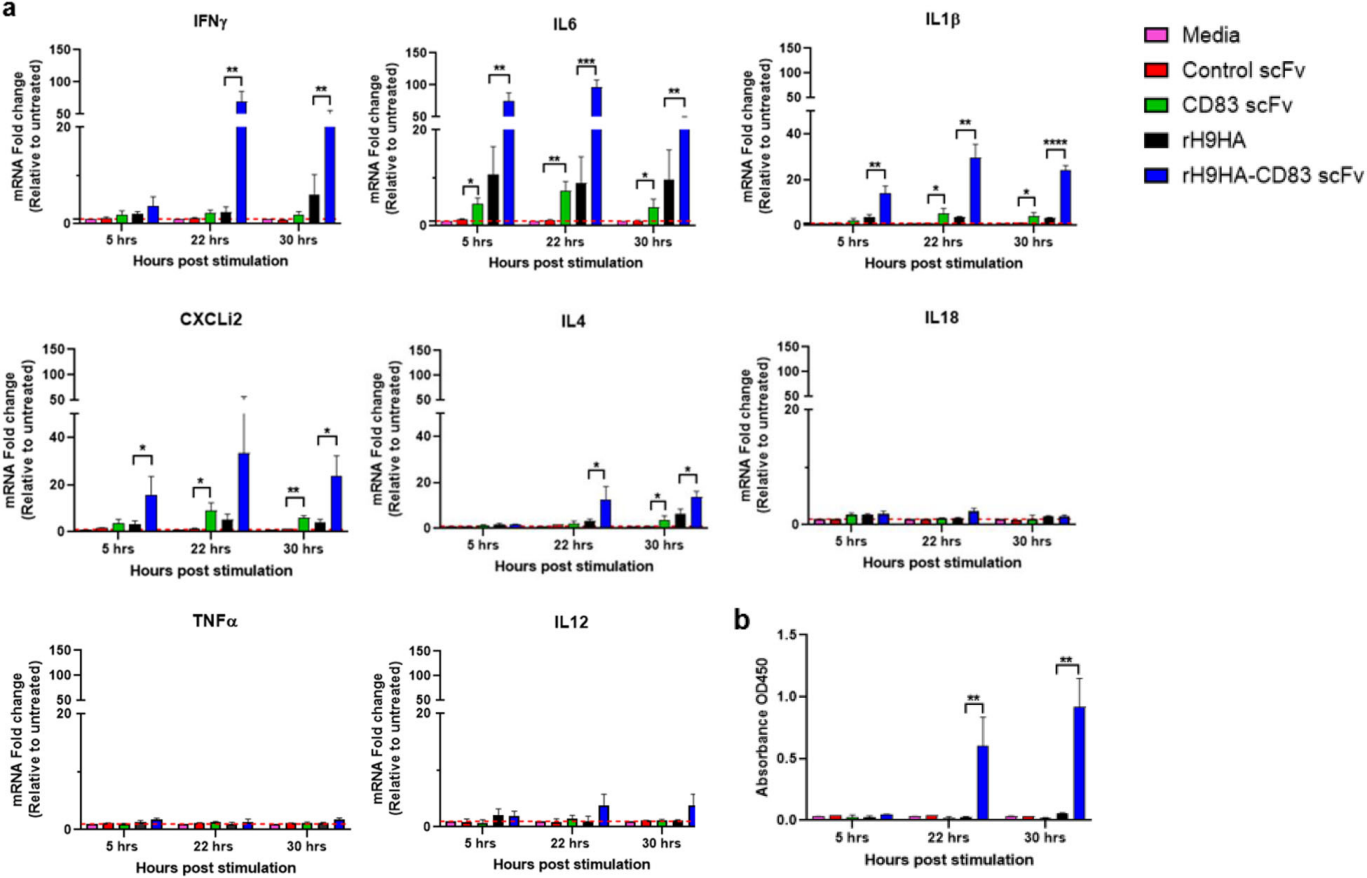
Analysis of cytokine (IFNγ, IL6, IL1β, IL4, IL18, TNFα, and IL12) and chemokine (CXCLi2) mRNA levels in splenocytes upon stimulation with CD83 scFv, rH9HA, and rH9HA-CD83 scFv. **a** Cytokines and chemokine mRNA levels in splenocytes analysed using qRT-PCR. **b** IFNγ protein level analysed using ELISA. For **a**: splenocytes were isolated from the spleen of 3-week-old SPF chickens using Histopaque 1083 and stimulated with 10 μg of CD83 scFv/rH9HA/rH9HA-CD83 scFv for 5, 22, and 30 h in vitro. Stimulated splenocytes were harvested for RNA extraction and expression levels of the respective cytokines and chemokine were measured by qRT-PCR. Data were calculated using 2^**−∆∆CT**^ approach (*n*-fold change compared to the media only control group) and reported as values normalised to the expression level of a housekeeping gene RPLPO1. For **b**: supernatants from the stimulated splenocytes were analysed for the presence of IFNγ by ELISA. Data are represented as mean ± SD and analysed by one-way ANOVA followed by Tukey’s multiple comparison test. ***p < 0.001 **p < 0.01 *p < 0.05. The data represent three independent experiments.

Splenocytes were isolated from specific-pathogen-free (SPF) chickens and treated with CD83 scFv, rH9HA, and rH9HA-CD83 scFv proteins for 5, 22, and 30 h in vitro. The production of different cytokines such as IFNγ, IL1β, IL6, IL4, IL18, TNFα, and IL12, as well as the chemokine CxCLi2, was investigated to examine the immunogenic potential of our vaccine candidates; rH9HA and rH9HA-CD83 scFv. With regards to stimulation by CD83 scFv, CD83 scFv was able to induce significantly higher mRNA levels of IL6, IL1β, CxCLi2, and IL4 (*p* < 0.05) compared to the control scFv at some time points (Fig. 4a). There was little or no induction of IFNγ, IL18, TNFα, and IL12. For IFNγ, verification was also carried out using IFNγ ELISA (Fig. 4b).

Interestingly, rH9HA-CD83 scFv stimulation showed an overall higher mRNA level of most of the cytokines tested compared to CD83 scFv. A significantly higher mRNA level of IFNγ, IL6, IL1β, CxCLi2, and IL4 (*p* < 0.05) was observed compared to rH9HA (Fig. 4a). There was little or no induction of IL18, TNFα, and IL12. Furthermore, IL6, IL1β, and CxCLi2 cytokines were induced earlier (5 h post stimulation) whereas IFNγ and IL4 were induced later (after 22 h post stimulation). Moreover, the expression of IFNγ in the supernatant after rH9HA-CD83 scFv induction was also verified using IFNγ ELISA assay (Fig. 4b). We also assessed the ability of rH9HA and rH9HA-CD83 scFv to stimulate chicken bone marrow-derived dendritic cells (BMDCs) for the production of different cytokines. The rH9HA-CD83 scFv was able to induce higher mRNA levels of the tested cytokines compared to rH9HA (Supplementary Fig. 1). This overall result suggests that rH9HA-CD83 scFv can promote the stimulation of chicken immune cells in vitro.

### Immunisation with rH9HA-CD83 scFv proteins induces a faster and higher humoral response

**Fig. 5:**
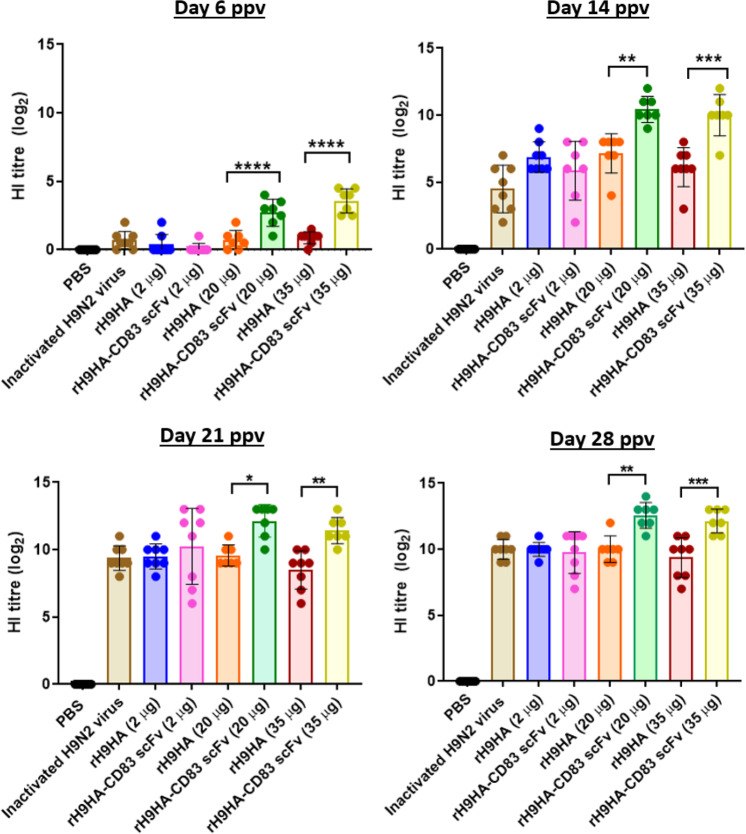
Analysis of HI antibodies in the serum of chickens vaccinated with rH9HA, rH9HA-CD83 scFv, and inactivated H9N2 virus. Groups of 7 days old chickens (*n* = 8) were immunised with 2.8, 28, and 49 µg of recombinant rH9HA-CD83 scFv equivalent to 2, 20, and 35 µg of recombinant rH9HA (equimolar concentration). Boost vaccination was given after 7 days post primary vaccination. The chickens were bled on day 6, 14, 21, and 28 posts primary vaccination. Antibody titres in sera were measured by haemagglutinin inhibition (HI) assay. The highest dilution of serum inhibiting the agglutination of RBCs by H9N2 virus (UDL01/08) was recorded. Data are presented as mean ± SD and analysed by one-way ANOVA followed by Tukey’s multiple comparison test. ****p < 0.0001 ***p < 0.001 **p < 0.01 *p < 0.05. ppv post primary vaccination.

A standard haemagglutination inhibition (HI) test was conducted to test the ability of rH9HA-CD83 scFv to generate H9HA specific antibodies. The serum antibody titres were measured at 6,14, 21, and 28 days post primary vaccination (ppv). With the 20 and 35 µg dose of rH9HA-CD83 scFv significantly higher amount of H9HA specific HI antibodies (*p* < 0.0001) were observed as early as day 6 ppv compared to the respective concentrations of rH9HA (Fig. 5). The HI antibody response induced by the 2 µg dose of rH9HA-CD83 scFv was slower when compared to that induced by 20 and 35 µg doses. However, after 14 days ppv even the 2 µg rH9HA-CD83 scFv vaccine group had HI antibody titre similar to the inactivated vaccine group. Furthermore, with the 20 and 35 µg doses of rH9HA-CD83 scFv vaccine group, the titre of HI antibodies produced was higher than rH9HA vaccine group at all the time points tested. Interestingly, there were no significant differences between the 2, 20, and 35 μg doses in rH9HA immunised groups at all the time points tested. However, with rH9HA-CD83 scFv significantly higher HI antibodies were produced with the 20 and 35 μg doses compared to the 2 μg dose on most of the time points tested (*p* < 0.0001 for day 6 ppv, *p* < 0.001 for day 14 ppv, *p* < 0.01 for day 28 ppv). Furthermore, the HI antibody titres with 20 μg and 35 μg of rH9HA-CD83 scFv were also significantly higher than that of the inactivated H9N2 virus vaccine group (*p* < 0.001 for day 6 ppv, *p* < 0.001 for day 14 ppv, *p* < 0.01 for day 28 ppv).

**Fig. 6:**
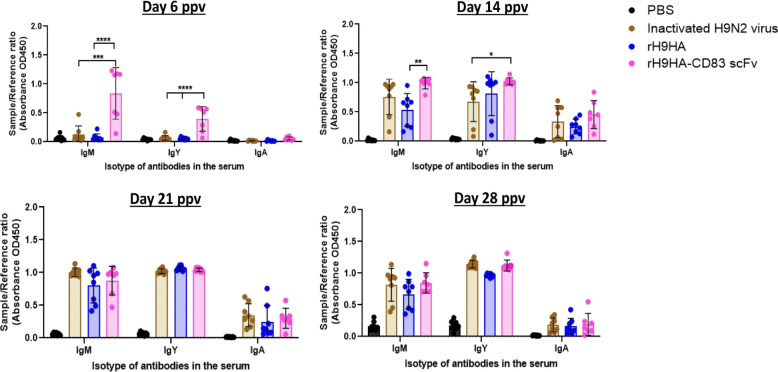
HA-specific IgM, IgY, and IgA antibody levels in the serum of chickens immunised with 35 μg dose of rH9HA, rH9HA-CD83 scFv, and inactivated H9N2 virus vaccines. The HA-specific isotypes of the antibodies were determined in 200-fold diluted sera collected at day 6, 14, 21, and 28 post primary vaccination by ELISA. The plates were coated with 1 μg of recombinant H9HA protein overnight at 4 °C. For detection, the plates were incubated with respective sera for 1 h at room temperature. This was followed by further incubation for 1 h with 1:3000 diluted anti-chicken IgM, IgY, and IgA antibodies. The colorimetric detection was carried out by adding tetramethylbenzidine (TMB) substrate and absorbance at 450 nm was recorded. The amount of HA-specific IgM, IgY, and IgA antibodies were expressed as a sample to reference ratio (relation of absorbance of a tested serum sample to the absorbance of the reference serum). Data are presented as mean ± SD and analysed by one-way ANOVA followed by Tukey’s multiple comparison test. ****p < 0.0001 ***p < 0.001 **p < 0.01 *p < 0.05. ppv post primary vaccination.

An ELISA approach was used to determine the total anti-HA reactivity for different classes of antibody (IgM, IgY, and IgA) in the serum of the immunised chickens with 35 μg dose of vaccines at 6, 14, 21, and 28 days ppv. As expected^27^, the amount of anti-HA IgM and IgY antibodies were higher than IgA antibodies in the immunised serum (Fig. 6). On day 6 ppv the amount of anti-HA IgM and IgY antibodies were significantly higher in rH9HA-CD83 scFv group compared to rH9HA and inactivated H9N2 virus vaccine groups (*p* < 0.001). Furthermore, higher anti-HA IgM antibodies were also observed in rH9HA-CD83 scFv group compared to rH9HA group on day 14 ppv (*p* < 0.01). Moreover, no differences were observed in the amount of anti-HA IgY antibodies between rH9HA and rH9HA- CD83 scFv groups after day 14 ppv.

**Fig. 7:**
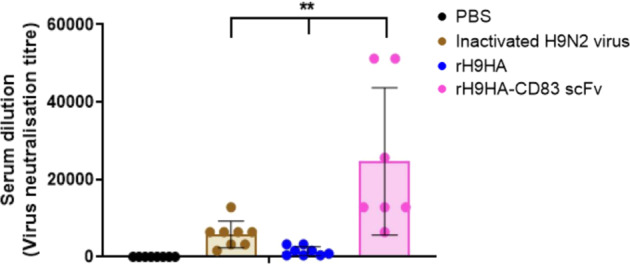
Virus neutralising antibody titre measured by virus micro-neutralisation (MNT) test. Virus MNT was carried out on day 28 post primary vaccination serum samples (35 μg dose group). The serum was first inactivated at 56 °C for 30 min and two-fold serum dilutions were made in phosphate-buffered saline. Then, the inactivated serum was incubated with 150 TCID_50_ of H9N2 virus (UDL 01/08) for 1 h at 37 °C. The virus-serum mixture was then added onto the MDCK cells and further incubated for 1 h at 37 °C. After 1 h of incubation, the virus-serum mixture was removed from the cells and fresh media containing 2 μg/ml trypsin was added. The cells were stained with 0.1% crystal violet solution after 3 days. The virus neutralisation titre was expressed as the reciprocal of the highest serum dilution at which virus infection is blocked and the cells survive. Data are presented as mean ± SD and analysed by one-way ANOVA followed by Tukey’s multiple comparison test. **p < 0.01.

To determine the effect of serum antibodies on viral biology, virus micro-neutralisation (MNT) test was also performed with immunised sera from 35 μg vaccine dose at 28 days ppv. The MNT assay is believed to be more sensitive than the HI assay, and it assesses the ability of the serum antibodies to block virus entry and productive replication^28^. The virus neutralisation antibody titre of rH9HA-CD83 scFv group was significantly higher than rH9HA and inactivated H9N2 virus vaccine groups (*p* < 0.01, Fig. 7).

### rH9HA-CD83 scFv is better at reducing the viral load in H9N2 virus challenged chickens compared to rH9HA

To determine the protective efficacy of rH9HA-CD83 scFv vaccine, groups of chickens were vaccinated twice (day 7 and day 14 old) with (i) rH9HA (ii) rH9HA-CD83 scFv (iii) inactivated H9N2 virus and (iv) phosphate-buffered saline (PBS, negative control), and challenged with A/chicken/Pakistan/UDL 01/2008 (UDL01/08) H9N2 virus after 7 days post boost vaccination (day 22 old). The PBS treated and rH9HA-CD83 scFv vaccinated groups also had un-infected chickens serving as contacts. The contact groups provide evidence on whether the vaccinated chickens have a reduced chance of getting an infection from the directly infected PBS control chickens while sharing the same air space, food, and water.

**Fig. 8:**
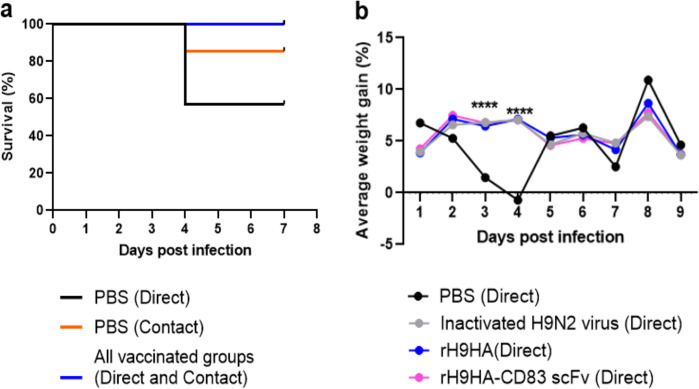
Survival and average weight gain of chickens after virus challenge. **a** Percentage survival between vaccinated and PBS treated control chickens challenged with H9N2 virus. Directly infected and contact birds were monitored for clinical disease signs throughout the study duration. Birds that reached the humane endpoints were culled due to ethical reasons. Survival curves between directly infected PBS control group (black line) and directly infected or contact vaccinated groups (blue line) significantly different *P-*value ≤ 0.05 (log-rank (Mantel−Cox) test). **b** Percentage average weight gain per day between vaccinated and PBS treated control chickens challenged with H9N2 virus. Chickens were weighted daily during the study duration. Data are analysed by one-way ANOVA followed by Tukey’s multiple comparison test. Statistical significance between the vaccinated and PBS treated control groups is shown with asterisks ****p < 0.0001.

The clinical signs observed in the virus-infected chickens include diarrhoea, rapid breathing, weight loss, eyes half shut, ruffled feathers, depression, and isolated behaviour. All vaccinated groups (direct and contact) had 100% survival rates compared with 58% in the directly infected PBS control group. In addition, the survival rate of the PBS treated contact group chickens was 87% (Fig. 8a). The average weight gain of all directly infected vaccinated chickens remained consistent, whereas the average weight gain in directly infected PBS control group chickens was significantly decreased on day 3 and day 4 post virus infection (*p* < 0.0001 for both days, Fig. 8b). Three chickens in the directly infected PBS control group showed severe clinical disease signs and were humanely culled due to ethical reasons on day 4 post virus infection. The chickens that survived had a similar average weight gain pattern as the vaccinated groups.

**Fig. 9:**
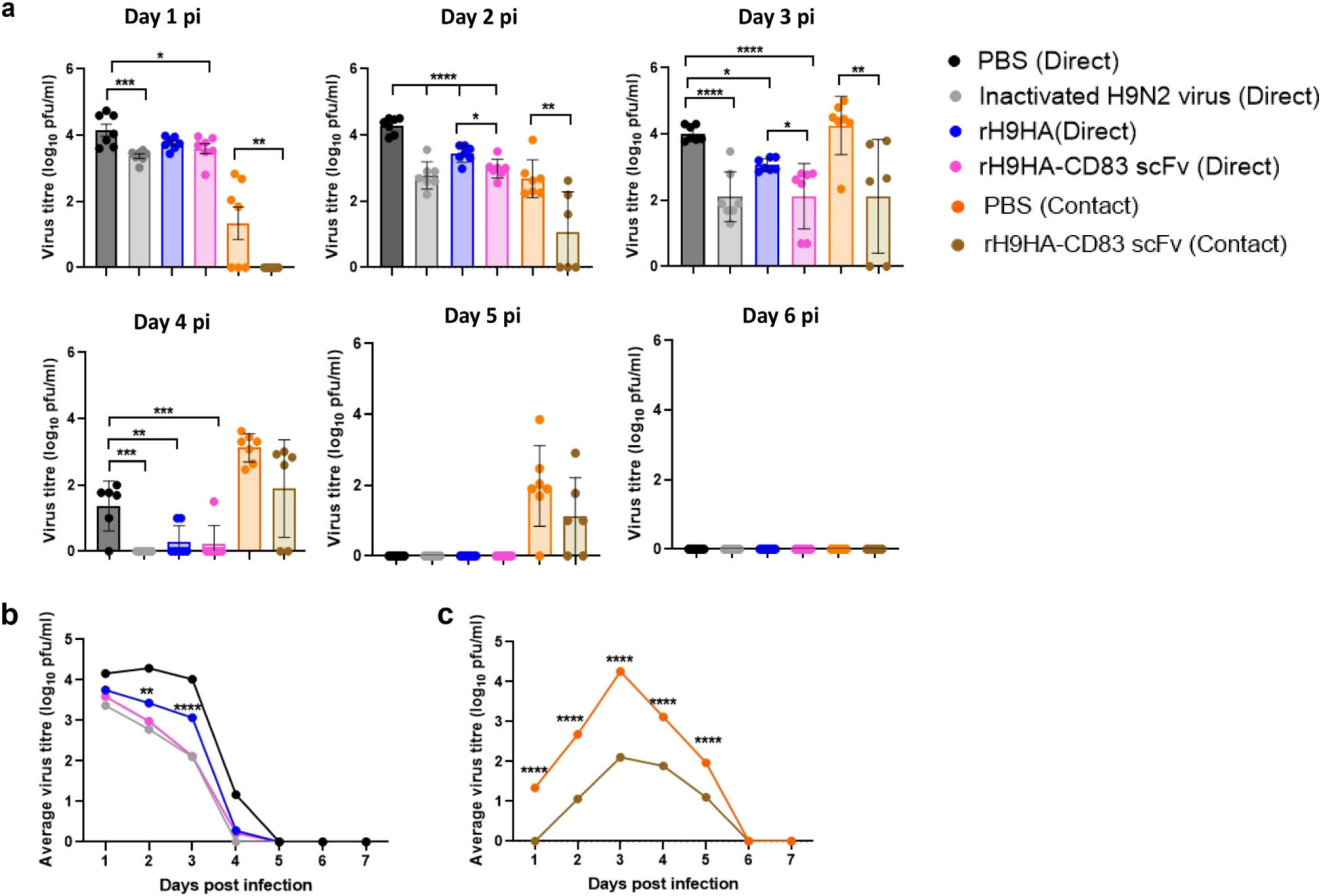
Challenge virus shedding profiles from the buccal cavity of vaccinated and PBS treated control chickens infected with H9N2 virus (UDL 01/08). Groups of 7-day-old chickens (*n* = 7) were immunised with 35 µg of recombinant rH9HA-CD83 scFv equivalent to 25 µg of recombinant rH9HA (equimolar concentration). The second vaccine dose as boost was given on day 7 post primary vaccination. The chickens were challenged with 100 μl of 1 × 10^7^ pfu/ml H9N2 virus (UDL 01/2008) after 7 days post boost vaccination. Some of the vaccinated and control birds were left unchallenged to serve as contacts. Swabs were taken from the buccal cavity until day 7 post infection and the amount of live virus particles recovered from swabs was titrated via plaque assays and presented as plaque-forming units (PFU)/ml. **a** Shedding profiles of each chicken from the buccal cavity at different days post infection are indicated by a single coloured dot. **b** The average shedding profile from the buccal cavity per group for directly infected birds. **c** The average shedding profile from buccal cavity per group for contact birds. Data are presented as mean ± SD for (**a**) and analysed by one-way ANOVA followed by Tukey’s multiple comparison test for (**a**) and by unpaired t-test for (**b, c**). For (**b**) the asterisks represent significant difference between rH9HA and rH9HA-CD83 scFv (direct) groups. ****p < 0.0001 ***p < 0.001 **p < 0.01 *p < 0.05. pi post infection.

The viral load in chickens was determined by plaque assays of buccal and cloacal swabs collected from day 1 to day 7 post infection. No cloacal virus shedding was observed in any chickens challenged with H9N2 virus (data not shown). Viral titres in the buccal swabs collected from vaccinated groups on day 1, 2, 3, and 4 post infection remained significantly lower compared to the PBS control group (Figs. 9a, b). The average viral load, in directly infected PBS control groups, ranged from 23 000 to 12 000 pfu/ml on the first three days post virus infection, whereas for vaccinated groups the average viral load ranged from 6200 to 390 pfu/ml. Furthermore, the rH9HA-CD83 scFv vaccinated chickens had significantly lower average viral load compared to rH9HA vaccinated chickens on day 2 post infection (rH9HA-CD83 scFv: 1220 pfu/ml, rH9HA: 3052 pfu/ml) and day 3 post infection (rH9HA-CD83 scFv: 390 pfu/ml, rH9HA: 1257 pfu/ml). There were not any significant differences between rH9HA-CD83 scFv and inactivated virus vaccine groups in terms of viral load. By day 4 post infection, the virus was almost cleared from all the directly infected vaccinated groups, whereas some virus remained detectable (<2 log_10_ pfu/ml) in the directly infected PBS control group. In addition, the rH9HA-CD83 scFv contact group chickens had significantly lower viral load compared with those in the PBS contact group (Fig. 9a, c). These results clearly demonstrate that the rH9HA-CD83 scFv induced a strong HA-specific antibody response that resulted in reduced clinical disease signs, virus shedding, and mortality.

## Discussion

Vaccines are key to protect poultry against pathogens for reasons of welfare, production, and reduced risk of zoonotic infection in humans. There is a critical need to improve the immunogenicity of poultry vaccines and one strategy is based upon enhancing delivery to professional APCs, such as dendritic cells, macrophages, and B cells. Here, we designed and developed a vaccine based on targeted delivery of the HA antigen of H9N2 influenza virus. The H9HA was produced as a trimeric soluble glycoprotein and recombinantly fused with an scFv portion of an antibody that is specific for the chicken CD83 molecule. Best characterised in mice and humans, CD83 is most highly expressed on mature DCs but can also be expressed on other cell types including B cells, macrophages, and NK cells^21^. The soluble H9HA protein was produced by eliminating the transmembrane domain and fusing the C-terminus of H9HA protein to trimerisation foldon of T4 bacteriophage. The fusion of H9HA protein with CD83 scFv antibody does not seem to abrogate the functional activity of both HA as well as the scFv antibodies. The recombinant rH9HA-CD83 scFv produced in *Drosophila* S2 cells and secreted into the culture medium retained functional activity to agglutinate RBCs in HA assay and the CD83 scFv antibody fused to HA was able to detect and bind to chicken CD83 ED in ELISA assay.

The vaccination of chickens with H9HA antigen carrying CD83 receptor-specific scFv induced faster and stronger immunity compared to the H9HA antigen lacking CD83 receptor-specific scFv. The mechanism by which rH9HA-CD83 scFv triggered this enhancement is not fully clear. It is surprising that high antibody responses were observed in the absence of DC-activating adjuvants like LPS and CpG oligonucleotides, suggesting that the side effects of these adjuvants could be avoided by using CD83 scFv targeted vaccine. Studies have shown that anti-CD83 antibodies have immunosuppressive potential thus, they can be used for the treatment of acute graft-versus-host disease (GVHD), which occurs after allogenic hematopoietic stem cell transplant due to the cascades of inflammatory reactions of donor immune cells^29,30^. Here, we evaluated the potential enhancing effects of CD83 scFv and rH9HA-CD83 scFv in vitro on chicken splenocytes, by examining the cytokine mRNA expression levels. CD83 scFv on its own induced moderate upregulation of mRNA encoding pro-inflammatory cytokines i.e., IL6, IL1β, and CxCLi2, but no IFNγ. Interestingly, when CD83 scFv was fused to rH9HA, we observed higher upregulation of mRNA encoding IFNγ, along with other cytokines like IL6, IL1β, CxCLi2, and IL4. It has been suggested that antigen binding to the antibody could result in conformational changes in the antibody. This can enhance the ingestion of antigen-antibody complexes by Fc receptor-containing cells compared to the free antibody^31^. In our case, we have used scFv antibodies lacking Fc region. However, H9HA protein fusion to CD83 scFv antibodies could have induced some structural changes, allowing the rH9HA-CD83 scFv complex to be readily uptaken by the APCs and thus, increasing the immunostimulatory potential of the antigen.

The results from the in vivo dose titration vaccination study suggested that the serum HI antibody titres were higher for chickens vaccinated with 20 and 35 µg rH9HA-CD83 scFv compared to rH9HA. Furthermore, the HI antibodies were produced as early as 6 days ppv with 20 and 35 µg doses of rH9HA-CD83 scFv compared to rH9HA. Thus, we predict that the optimal vaccine dose for faster and higher humoral response with rH9HA-CD83 scFv could be between 20 and 35 µg although, chickens vaccinated with 2 µg displayed similar HI antibody titres by 21 days ppv. An earlier study in which a complete HA protein was chemically conjugated with Dec205 mAb reported an increased serum antibody titre with 50 and 100 µg per dose of the targeted protein^10^. In our study, we observed a good antibody response with lower doses of CD83 scFv targeted H9HA vaccine (20 µg/35 μg). This provides an additional advantage of dose sparing. However, only a single dose of vaccine was used in the former study, whereas in this study, two doses of vaccine were used. Furthermore, the second vaccination was given just seven days after the first vaccination. It has been shown that an interval of several weeks between the first and second vaccination is important to obtain optimal immune responses^32^. Therefore, it is possible that our vaccination regime might not have given us the maximum immune responses. Several studies have reported that inactivated virus vaccines are better at evoking higher HI antibodies compared to subunit vaccines^33–37^. In this study, we observed that recombinant rH9HA-CD83 scFv produced higher serum HI and virus neutralising antibodies compared to the inactivated virus vaccine. It has been reported that anti-CD83 mAb can have an adjuvant effect by modulating B cell function^38,39^. Mice injected with nitroiodophenol conjugated to Ficoll (NIP-Ficoll) along with anti-CD83 mAb showed a dramatic increase in the production of NIP-specific IgG1 antibodies, indicating that the ligation of anti-CD83 mAb to naturally induced CD83 receptor protein strongly affects antigen-specific IgG responses of B cells in vivo^39^. This could be a potential reason for the enhancement of antibody response by CD83 scFv targeted H9HA subunit vaccine. Some mammalian APC targeting studies have shown to induce both humoral and T cell-mediated immunity (CMI)^40,41^. We assessed CMI induced by rH9HA and rH9HA-CD83 scFv by using IFNγ ELISPOT. However, due to the lack of optimal recall antigen, no CMI was observed with IFNγ ELISPOT assay (data not shown). In addition, we also tested in vivo IFNγ response using RNA from the vaccinated spleens on day 2, 4, 6, 8, and 21 ppv, but failed to detect any upregulation (Supplementary Fig. 2). Therefore, further studies are needed to investigate the CMI elicited by rH9HA-CD83 scFv.

In terms of protection, rH9HA-CD83 scFv was better at reducing the viral load in the H9N2 virus-challenged chickens compared to rH9HA, at a level comparative to the inactivated virus vaccine. With the inactivated virus vaccine, antibodies are produced not just for HA protein but also for other viral proteins like neuraminidase and matrix protein 2. It is therefore promising that the recombinant rH9HA-CD83 scFv vaccine induced a similar level of protection to the industry standard inactivated H9N2 virus vaccine. One of the key characters of the inactivated vaccines is that the viral material also includes a range of products capable of stimulating innate immunity via pattern recognition receptor-based activation of immune cells such as DCs^34,35^. This activating character is often not as strong with recombinant protein vaccines where it needs to be entirely delivered by the adjuvant carrier. In the case of the rH9HA-CD83 scFv vaccine, we were able to demonstrate activation of ex vivo splenocytes via the anti-CD83 effect to induce upregulation of mRNA encoding inflammatory cytokines (IFNγ, IL1β, and IL6) and the chemokine CXCLi2. All of these indicate activation of immune cells which could combine to enhance immune responses as indicated by the high levels of antibodies and protective effect detected with this vaccination strategy.

To conclude, this study exploits the specificity and high-affinity binding properties of antibodies to create TADV for avian influenza in poultry. The approach using CD83 targeting led to high levels of effective antibodies (as measured by HI and MNT) and detectable antigen-specific responses with all classes of antibody in the chicken (IgM, IgY, and IgA). Most importantly, the recombinant rH9HA-CD83 scFv was effective at reducing the viral load in chickens to levels greater than rH9HA and equivalent to the industry standard inactivated virus approach. This CD83 targeting approach represents a powerful addition to the armoury of the developers of poultry vaccines reducing the need to grow large quantities of live virus for inactivated AIV vaccines. A similar approach could also be used in the development of safe, effective vaccines against other poultry and livestock diseases.

## Methods

### Ethics statement

All animal studies and procedures were carried out in strict accordance with the guidance and regulations of European and United Kingdom Home Office regulations under project licence number P68D44CF4. All animal work was reviewed and approved by the Animal Welfare Ethical Review Body at The Pirbright Institute.

### Viruses and cells

A/Chicken/Pakistan/UDL 01/2008 (UDL01/08) H9N2 virus was propagated in 10-day-old SPF embryonated hens’ eggs and titrated by plaque assay or TCID_50_ (50% tissue culture infective dose) on Madin Darby canine kidney (MDCK) cells. The virus was inactivated chemically using 0.1% beta-propiolactone (BPL, Alfa Aesar) and purified by ultracentrifugation through a continuous 30−60% w/v sucrose gradient.

MDCK cells were maintained in Dulbecco modified eagle medium (DMEM, Merck Life Science) supplemented with 10% fetal bovine serum (FBS) and 0.1% penicillin and streptomycin at 37 °C, 5% CO_2_. *Drosophila melanogaster* Schneider 2 (S2) cells were obtained from Thermofisher Scientific and maintained in Schneider’s insect medium (Merck Life Science) supplemented with 10% FBS at 25 °C.

### Construction of CD83 scFv and H9HA fused CD83 scFv protein-expressing plasmids

The mouse hybridoma clone of chicken CD83 (clone: IAH F890:GE8) was produced at the Institute for Animal Health (now The Pirbright Institute) by immunising mice with chicken CD83 antigen (GenBank accession number: XM_040663657.1)^42^. The variable light (vL) chain and variable heavy (vH) chain sequences were derived from the hybridoma clones commercially (Absolute Antibody Ltd). Synthetic cDNA containing the vL and vH sequences of APC-mAb were joined by (Gly_4_Ser)_4_ linker peptide sequence and manufactured commercially by Geneart (Thermo Fisher Scientific). The respective individual vL-Linker-vH cDNA were then cloned into *Drosophila melanogaster* expression vector (pMT-BIP-V5-His version A, Thermo Fisher Scientific) using the *Not I* and *Xba I* restriction sites (Supplementary Figure 2). The resultant vector named pMT-BIP-CD83 scFv-V5-His was used to insert ectodomain of H9HA gene that lacks both HA gene signal peptide and the TM domain, replaced with a 30 amino acid trimerisation foldon sequence of the trimeric protein fibritin from bacteriophage T4, using *Kpn I* and *Pac I* restriction sites (Fig. 1b). The HA used in this study was synthetically produced incorporating consensus sequence of HA of H9N2 viruses derived from analysis of over 2000 H9HA sequences (from the public database) of G1-like H9 virus lineage using Minimum Sphere Consensus (MScon) method (developed by Jan Kim, The Pirbright Institute, UK). This synthetic HA has 98% amino acid sequence similarity to HA ectodomain of H9N2 virus (UDL01/08) (GenBank accession number: ACP50708.1, HA1: 19-338, and HA2: 339-560).

### Expression and purification of CD83 scFv and H9HA fused CD83 scFv proteins

Recombinant proteins were produced and purified using *Drosophila* Expression System (DES^®^, Life technologies). Briefly, pMT-BIP-CD83 scFv-V5-His, pMT-BIP-rH9HA-V5-His, or pMT-BIP-rH9HA-CD83 scFv-V5-His plasmids were co-transfected into *Drosophila melanogaster* S2 cells together with a hygromycin B resistance plasmid (pCoHYGRO, Life technologies). Antibiotic selection was carried out for four weeks using hygromycin B at a concentration of 250 µg/ml and a single cell clone was obtained via limiting dilution^43^. Recombinant proteins were secreted into culture supernatant after CuSO_4_ (500 µM) induction and then purified by Profinity™IMAC uncharged column (Bio-Rad). Protein expression was determined by sodium dodecyl sulfate–polyacrylamide gel electrophoresis (SDS-PAGE) using 10−12% gradient polyacrylamide gels (Bio-Rad) followed by Coomassie staining (Life Technologies). Furthermore, proteins were also transferred onto nitrocellulose membranes (Amersham) for western blotting using anti-H9HA monoclonal antibodies^44^. The concentration of purified recombinant proteins was determined by Pierce™ BCA Protein Assay Kit (Life Technologies) according to the manufacturer’s protocol.

### Characterisation of CD83 scFv and rH9HA-CD83 scFv proteins

Indirect ELISA was carried out to examine if CD83 scFv and rH9HA-CD83 scFv proteins can detect and bind to chicken CD83 ectodomain. The coding sequence of the chicken CD83 ectodomain^42^ was cloned into pMT-BIP-His vector for expression in *Drosophila melanogaster* S2 cells. Briefly, 8 μg of the respective receptor proteins were added onto the first well of 96 well maxisorp ELISA plates (Thermo Fisher Scientific). Then, a two-fold dilution of the respective receptor proteins was made in carbonate buffer (15 mM NaCO_3_, 35 mM NaHCO_3_, 3 mM NaN_3_). The plates were incubated at 4 °C overnight. For detection, the plates were incubated with equimolar concentration of the CD83 scFv and rH9HA-CD83 scFv or 1 μg/ml of CD83 mAb for 1 h at 4 °C. This was followed by further incubation with horseradish peroxidase-conjugated (HRP) anti-V5 secondary antibody (Bio-Rad Antibodies, primary antibody: CD83 scFv or rH9HA-CD83 scFv) or goat anti-mouse HRP secondary antibody (Bio-Rad Antibodies, primary antibody: CD83 mAb). The colorimetric detection was carried out by adding Tetramethylbenzidine (TMB) substrate (Thermo Fisher Scientific) and read at wavelength 450 nm in ELx808 absorbance microplate reader (BioTek).

### Bis[sulfosuccinimidyl] suberate (BS3) crosslinking

To determine the oligomeric structure of the recombinant H9HA containing trimerisation foldon domain, cross-linking was performed using BS3 (Thermofisher Scientific) as described by Weldon et al.^45^ with the following modifications. Briefly, 15 µg recombinant protein was incubated at room temperature in the presence of BS3 (final concentration 10 mM) for 1 h. Cross-linking was stopped by the addition of 1 M Tris-HCl pH 8.0 to a final concentration of 50 mM. The cross-linked products were separated on SDS gel under reducing conditions, blotted, and immunodetected using anti-H9HA monoclonal antibody^44^.

### Preparation and stimulation of chicken splenocytes

Splenocytes were prepared from the spleens of 3-week-old SPF chickens (Rhode Island Red, Roslin) via density gradient centrifugation by using Histopaque 1083 (Merck Life Science) according to the manufacturer’s instructions. About 2 × 10^6^ cells were plated on each well of a 24-well plate suspended in 300 μl of complete Roswell Park Memorial Institute 1640 (RPMI) medium containing 10% FBS and 0.1% penicillin and streptomycin. Cells were treated with 10 μg of rH9HA or 14 μg of rH9HA-CD83 scFv (containing 10 μg rH9HA according to the molecular weight) or 10 μg of CD83 scFv or 10 μg of control scFv (anti-H9HA). All cells were stimulated for 5, 22, and 30 h in vitro at 41 °C.

### RNA extraction and quantitative reverse transcription PCR (qRT-PCR) of cytokines and chemokines

RNA was extracted from the stimulated splenocytes using RNeasy kit (Qiagen) according to the manufacturer’s protocol. To perform RNA quantification, single-step real-time reverse transcription PCR was done using Superscript III Platinum One-Step qRT-PCR kit (Life Technologies) as per the manufacturer’s protocol in 7500 fast real-time PCR machine (Applied Biosystems). Cycling conditions include: (i) 50 °C for 5 min, (ii) 95 °C for 2 min and (iii) 40 cycles of denaturation at 95 °C for 3 s followed by annealing and extension at 60 °C for 30 s. Sequences of primers and probes used for qRT-PCR are shown in Supplementary Table 1. Cycle threshold (CT) values were obtained using 7500 software version 2.3. Data were calculated using the 2^**−∆∆CT**^ approach (*n*-fold change compared to the media only control group) and reported as values normalised to the expression level of a housekeeping gene RPLPO1 (Ribosomal phosphoprotein lateral stalk subunit PO). Out of the three reference genes (RPLPO-1, RPL13, and 28S) selected for normalisation, RPLPO1 was the most stable gene across samples and hence was chosen for normalisation.

### IFNγ sandwich ELISA

Supernatants from the stimulated splenocytes were harvested and examined by sandwich ELISA. Briefly, anti-chicken IFNγ (2 μg/ml, Invitrogen) was coated onto 96-well maxisorp ELISA plates (Thermo Fisher Scientific). The coated plates were blocked at room temperature with PBS containing 3% BSA for 1 h. A 1:2 dilution of the supernatants was made in PBS buffer containing 3% BSA. The plates were then incubated with the diluted supernatants for 2 h at room temperature. Detection was carried out using biotinylated anti-chicken IFNγ detection antibody (1 μg/ml, Invitrogen) for 1 h at room temperature followed by HRP conjugated streptavidin (1:1000 dilution, Amersham) for another 1 h at room temperature. A 100 μl of Tetramethylbenzidine (TMB) substrate (BD biosciences) was added for 10 min. The reaction was stopped using 2 M H_2_SO_4_ and read at wavelength 450 nm in ELx808 Absorbance Microplate Reader (BioTek).

### Haemagglutination assay and Haemagglutination inhibition assay

World Health Organisation guidelines were followed for HI assay^46^ and haemagglutination assay was performed as previously described^47^. For the HI assay, two-fold serial dilution of the serum was prepared by mixing 25 μl of serum with 25 μl PBS. Next, four HA units of the H9N2 virus (UDL01/08) were added to the diluted serum and incubated at 37 °C for 1 h. Finally, 50 μl of 1% chicken red blood cells were added onto the serum−virus mixture and incubated at room temperature for 45 min. HI titres were expressed as reciprocal of the highest dilution of antiserum that causes total inhibition of four HA units of virus hemagglutination activity.

### Chicken vaccination and blood sample collection

Groups of 7-day-old SPF chickens (Dekalb White, Henry Stewart & Co.Ltd) (*n* = 8 per group) were immunised with vaccine dose containing 2.8, 28, and 49 µg of recombinant rH9HA-CD83 scFv proteins equivalent to 2, 20, and 35 µg of recombinant rH9HA (equimolar concentration). The proteins were formulated in Montanide ISA 71 R VG (Seppic) as water-in-oil emulsion. The ratio of protein to adjuvant volume was 1:2. The vaccine dose (0.2 ml) was administered subcutaneously at the back of the neck. Control groups were immunised with PBS. Additionally, one group of chickens (*n* = 8) was vaccinated with inactivated H9N2 virus (UDL01/08, ~1 × 10^8^ EID_50_ (50% egg infectious dose) per ml). All vaccinated groups received a second booster dose at 14 days old. In all cases, blood samples were collected from the wing vein 6, 14, 21, and 28 days post primary vaccination.

### Viral challenge and swab sample collection

To evaluate the protective efficacy of vaccines against virus challenge, groups of 7-day-old SPF chickens (Rhode Island Red, from The National Avian Research Facility at Roslin Institute, University of Edinburgh, UK) were divided into groups (*n* = 7 per group) and each chicken was injected subcutaneously with 0.2 ml dose of vaccine or PBS (as control) at day 7 and 14 days of age. The vaccine groups include: (G1) inactivated H9N2 vaccine containing UDL 01/08 virus (~1 × 10^8^ EID_50_ per ml), (G2) rH9HA vaccine (25 μg/dose), (G3) rH9HA-CD83 scFv vaccine (25 μg/dose equivalent of rH9HA) and (G4) PBS control. Two additional groups (G5 and G6) were also included to serve as contacts that received PBS and rH9HA-CD83 scFv vaccine, respectively. Groups were housed in two separate isolators. The groups in isolator-1 include (G1) inactivated H9N2 virus (direct), (G2) rH9HA (direct), and (G3) rH9HA-CD83 scFv (direct). The groups in isolator-2 include (G4) PBS control (direct), (G5) PBS control (contact), and (G6) rH9HA-CD83 scFv (contact). All chickens in groups (G1−G4) except the contact groups (G5 and G6) were inoculated intranasally with 1 × 10^6^ plaque-forming units (PFU)/100 μl of H9N2 virus (UDL01/08) at day 22 old (14 days after the primary vaccination). Chickens were monitored daily for clinical signs and weight changes throughout the experiment.

Swab samples from buccal and cloacal cavities were collected daily until day 7 post infection with the last sampling performed on day 10 post infection. Sterile polyester tipped swabs were transferred into the virus transport media^48^, vortexed, and centrifuged for 10 min at 4500 rpm to clarify the medium. Samples were stored at −80 °C until further analysis.

### Measurement of serum IgM, IgY, and IgA anti-HA antibody levels

Antigen-specific IgM, IgY, and IgA antibody levels in the sera were determined by ELISA assay. Briefly, flat-bottom 96-well maxisorp ELISA plates (Thermo Fisher Scientific) were coated with 1 μg of recombinant rH9HA protein diluted in 50 μl of carbonate buffer (15 mM NaCO_3_, 35 mM NaHCO_3_, 3 mM NaN_3_) overnight at 4 °C. Protein-coated plates were blocked at room temperature with 5% milk powder (Marvel) in PBS-tween 0.1% (PBS-T) for 1 h. Plates were washed thrice with wash buffer PBS-T. Chicken sera was diluted to 1:200 in PBS-T buffer containing 1% skimmed milk powder (Marvel). The plates were then incubated with 50 μl of the diluted sera at room temperature for 1 h. Plates were again washed thrice and incubated for 1 h at room temperature with 50 μl of goat anti-chicken IgM (Life Technologies), IgY (Bio-Rad) and IgA (Life Technologies) antibody conjugated to HRP diluted in PBS-T buffer (1:3000 for IgM and IgA, 1:6000 for IgY) with 1% milk powder. Plates were washed ×4 with PBS-T then 100 μl of TMB substrate (BD biosciences) was added for 10 min. The reaction was stopped using 2 M H_2_SO_4_ and read at wavelength 450 nm in ELx808 Absorbance Microplate Reader (BioTek). A standard reference serum (serum collected from 35-day-old chicken (1:200 dilution) challenged with H9N2 virus (UDL01/08) was included in all assays. The amount of anti-HA IgM, IgY, or IgA antibodies were expressed as a sample to reference ratio (relation of absorbance of a tested serum sample to the absorbance of the reference serum).

### Plaque assay

The virus titre from the allantoic fluid or swab samples was obtained using plaque assay. Pre-seeded 12-well plates with MDCK cells were inoculated with ten-fold serially diluted sample in PBS and left for 1 h at 37 °C. Cells were washed with PBS and overlaid with DMEM (1× MEM, 0.21% BSA, 1 mM L-glutamate, 0.15% sodium bicarbonate, 10 mM Hepes, 0.1% penicillin G/streptomycin) containing 0.6% purified agar (Oxoid) and 2 µg/ml N-tosyl-L-phenylalanyl chloromethyl ketone (TPCK) trypsin (Merck Life Science). Cells were left at 37 °C for 72 h. After 3 days, culture media was removed, and cells were stained in crystal violet solution (Merck Life Science) for 30 min.

### Virus micro-neutralisation test

MDCK cells were pre-seeded into 96 well plates to reach 90−95% confluency. The immunised chicken sera were inactivated at 56 °C for 30 min. Then, 1:200 dilution of the inactivated serum was carried out in PBS. This was followed by a further two-fold serial dilution in triplicates and mixed with 90 μl of H9N2 virus (UDL01/08) containing 150 TCID_50_. The serum−virus mixture was incubated at 37 °C for 1 h. Cells were washed with PBS and inoculated with the serum−virus mixture for 1 h at 37 °C. After the incubation, cells were washed once again with PBS and serum-free DMEM containing 2 µg/ml TPCK trypsin was added, cells were left at 37 °C for 72 h. After 3 days the medium was removed, and cells were stained in 0.1% crystal violet solution for 30 min. Virus MNT titres were expressed as reciprocal of the highest dilution of antiserum that blocks the virus infectivity in cultured cells inoculated with 150 TCID_50_ (50% tissue culture infective dose).

### Statistical analysis

Results are expressed as mean ± standard deviation (SD). Statistical significance (*p*-values) was determined using a one-way ANOVA followed by post hoc Tukey’s multiple comparison test, log-rank (Mantel−Cox) test (survival plot), and unpaired Student’s t-test using Prism 8.3.0 (GraphPad Software). Differences were considered statistically significant if *p* < 0.05.

## Supplementary information

Supplementary Information

## Data Availability

Data that support the findings of this study are included in the article and supplementary information.
