# Author Correction: Selectively targeting haemagglutinin antigen to chicken CD83 receptor induces faster and stronger immunity against avian influenza

**DOI:** 10.1038/s41541-022-00579-6

**Published:** 2022-11-23

**Authors:** Angita Shrestha, Jean-Remy Sadeyen, Deimante Lukosaityte, Pengxiang Chang, Adrian Smith, Marielle Van Hulten, Munir Iqbal

**Affiliations:** 1 The Pirbright Institute, Pirbright, Woking, Surrey, United Kingdom; 2 Department of Zoology, Peter Medawar Building, University of Oxford, Oxford, United Kingdom; 3 Global Poultry R&D Biologicals Boxmeer, Intervet International BV, MSD Animal Health, Boxmeer, The Netherlands

**Keywords:** Vaccines, Vaccines, Virology

Correction to: *npj Vaccines* 10.1038/s41541-021-00350-3, published online 15 July 2021

In the original version of this Article, incorrect information about the supplier was included in the Methods for antibodies IgM, IgY and IgA. Instead of Abcam it should have been Life Technologies, Bio-Rad and Life Technologies, respectively. The HTML and PDF versions of the Article have now been updated with the correct information.

