# Supplementary material for: Selectively targeting haemagglutinin antigen to chicken CD83 receptor induces faster and stronger immunity against avian influenza
Source: NPJ Vaccines. 2021 Jul 15;6:90. doi: 10.1038/s41541-021-00350-3 (PMC8282863; doi:10.1038/s41541-021-00350-3)
Supplement: Supplementary file 1 — Supplementary Information [file 41541_2021_350_MOESM1_ESM.pdf]

Selectively Targeting Haemagglutinin Antigen to Chicken CD83 Receptor Induces Faster and Stronger Immunity against Avian Influenza

Supplementary information

Supplementary Table 1: Sequences of probes and primers used for qRT-PCR.

Gene	Primer sequences (5’-3’)	Probe sequence (5’-3’)
IFNγ	**F**: GTGAAGAAGGTGAAAGATATCATGGA	**FAM**-TGGCCAAGCTCCCGATGAACGA-**TAMRA**
	**R**: GCTTTGCGCTGGATTCTCA	
IL6	**F**: AACATGCGTCAGCTCCTGAAT	**FAM**- AGCAGCACCTCCCTCAAGGCACC-**TAMRA**
	**R:** TCTGCTAGGAACTTCTCCATTGAA	
IL1β	**F:** GCTCTACATGTCGTGTGTGATGAG	**FAM**-CCACACTGCAGCTGGAGGAAGCC-**TAMRA**
	**R:** TGTCGATGTCCCGCATGA	
IL4	**F:** AACATGCGTCAGCTCCTGAAT	**FAM**-AGCAGCACC TCCCTCAAGGCACC-**TAMRA**
	**R**: TCT GCTAGGAACTTCTCCATTGAA	
IL18	**F**: AGGTGAAATCTGGCAGTGGAAT	**FAM**-CCGCGCCTTCAGCACGGATG-**TAMRA**
	**R**: ACC TGGACGCTGAATGCAA	
TNFα	**F**: GACAGCCTATGCCAACAAGTA	**FAM-**TGTGTATGTGCAGCAACCCGTAGT-**TAMRA**
	**R:** TTACAGGAAGGGCAACTCATC	
IL12-p35	**F:** TGAAGGTGCAGAAGCAGAG	**FAM-**ACGTCACCAACAGTCAGAGCAACA-**TAMRA**
	**R:** CTGAGACGTGCAGGACTTTAT	
CxCLi2	**F**: GCCCTCCTCCTGGTTTCAG	**FAM**-TCTTTACCAGCGTCCTACCTTGCGACA-**TAMRA**
	**R:** TGGCACCGCAGCTCATT	
RPLPO-1	**F**: TTGGGCATCACCACAAAGATT	**FAM**-CATCACTCAGAATTTCAATGGTCCCTCGGG-**TAMRA**
	**R**: CCCACTTGTCTCCGGTCTTAA	

IFN: Interferon **IL**: Interleukin **TNFα**: Tumour necrosis factorCxCLi**2:** Chemotactic and angiogenic factor/IL8 **RPLPO-1**: Ribosomal phosphoprotein lateral stalk subunit PO F**:** Forward R: Reverse **FAM**: 6-carboxyfluorescein **TAMRA**: Tetramethylrhodamine

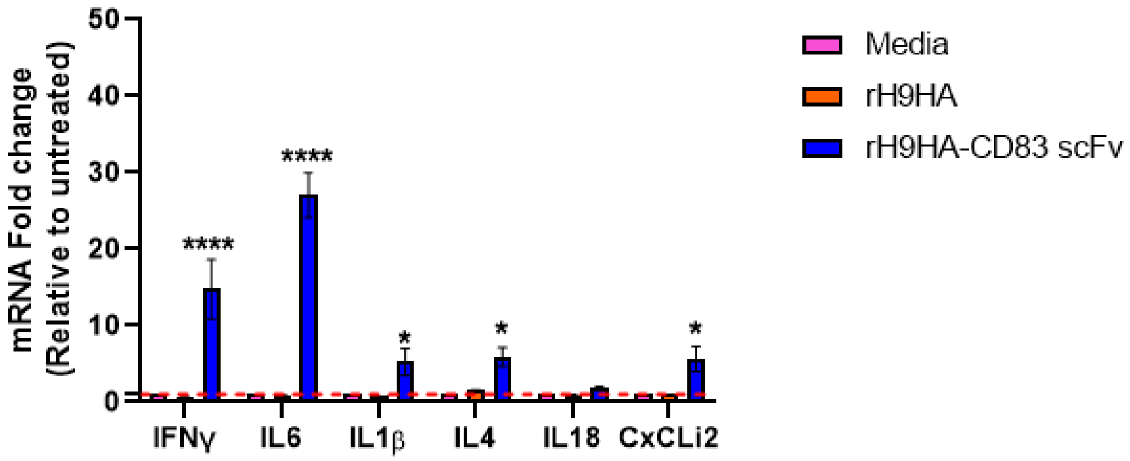

Supplementary Figure 1: qRT-PCR analysis of cytokines (IFNγ, IL6, IL1β, IL4, IL18) and chemokine (CXCLi2) production by chicken bone marrow derived DCs (BMDCs) upon stimulation with rH9HA and rH9HA-CD83 scFv. Chicken bone marrow cells were cultured in the presence of recombinant chicken granulocyte–macrophage colony-stimulating factor (GM-CSF) and recombinant chicken interleukin-4 (IL-4) for 7 days. BMDCs were stimulated with 10 µg of rH9HA/rH9HA-CD83 scFv for 22 hours *in vitro*. Stimulated BMDCs were harvested for RNA extraction and expression levels of the respective cytokines and chemokine were measured by qRT-PCR. Data were calculated using $2^{-\Delta\Delta CT}$ approach (n-fold change compared to the media only control group) and reported as values normalised to the expression level of a housekeeping gene RPLPO1. Data are presented as mean $\pm$ SD and analysed by one-way ANOVA followed by Tukey's multiple comparison test. ***p<0.001 **p<0.01 *p<0.05. The data represent three independent experiments.

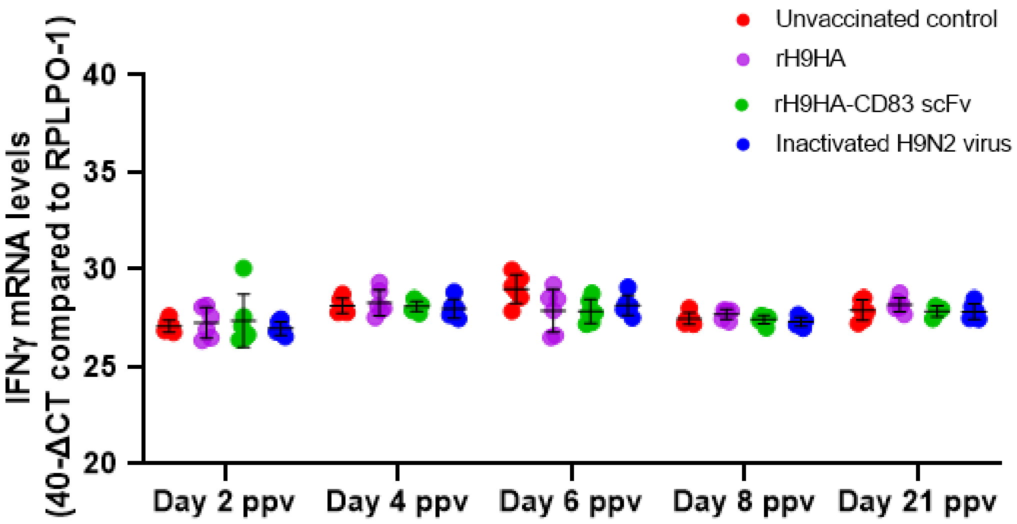

<u>Supplementary Figure 2</u>: Upregulation of IFNγ in the spleen of chickens vaccinated with rH9HA, rH9HA-CD83 scFv and inactivated H9N2 virus vaccines. Spleen samples were collected from vaccinated chickens on day 2, 4, 6, 8 and 21 post vaccination and RNA was extracted. qRT-PCR was performed for IFNγ and levels compared to RPLPO-1 reference gene were calculated as 40- ΔCT value. Data are presented as mean ± SD and analysed by one-way ANOVA followed by Tukey's multiple comparison test.

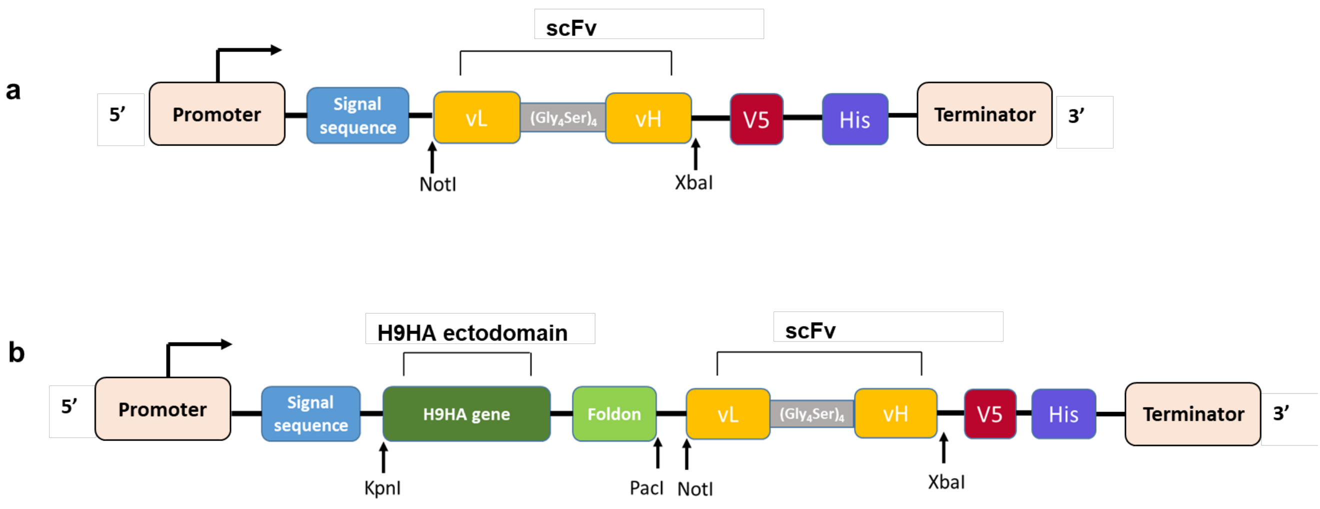

<u>Supplementary Figure 3</u>. Schematic representation of single chain fragment variable antibody (scFv) and H9HA ectodomain fused scFv antibody expression cassettes **(a)** The scFv expression cassette includes *Drosophila melanogaster* immunoglobulin heavy chain binding protein (BIP) secretion signal sequence at 5' end followed by APCs-specific mAb variable light chain (vL), linker peptide $(Gly_4Ser)_4$ and variable heavy chain (vH). The V5 tag is for detection and His tag for purification **(b)** H9HA Foldon-scFv fusion construct. The expression cassette includes BIP secretion signal sequence at 5' end followed by H9HA gene fused with HA trimerization signal (indicated as foldon), linked with APCs-specific mAb variable light chain (vL), linker peptide $(Gly_4Ser)_4$ and variable heavy chain (vH).
